# Is headache a risk factor for dementia? A systematic review and meta-analysis

**DOI:** 10.1007/s10072-023-07069-0

**Published:** 2023-09-18

**Authors:** Aurora Cermelli, Fausto Roveta, Lia Giorgis, Silvia Boschi, Alberto Grassini, Fabio Ferrandes, Chiara Lombardo, Andrea Marcinnò, Elisa Rubino, Innocenzo Rainero

**Affiliations:** 1 Headache Center, Department of Neuroscience, ‘‘Rita Levi Montalcini’’, University of Torino, Via Cherasco 15, 10126 Turin, Italy; 2 Department of Neuroscience and Mental Health, Città della Salute e della Scienza, Corso Bramante 88, Turin, Italy

**Keywords:** Primary headaches, Migraine, Dementia, Alzheimer’s disease, Association

## Abstract

**Objective:**

In this systematic review and meta-analysis, we critically evaluate available evidence regarding the association between primary headaches and subsequent decline of cognitive function and dementia.

**Background:**

Recent studies suggested that headache disorders may increase the risk for dementia. However, available studies are conflicting.

**Methods:**

To identify qualifying studies, we searched scientific databases, including *Pubmed*, *Scopus, Web of Science, Science Direct* and *BMC,* screening for relevant papers. In order to reduce the heterogeneity between different studies, the analyses were further subdivided according to the clinical diagnoses and the study methodologies.

**Results:**

We identified 23 studies investigating the association between primary headaches and the risk of dementia. Of these, 18 met our inclusion criteria for meta-analysis (covering 924.140 individuals). Overall effect-size shows that primary headaches were associated with a small increase in dementia risk (OR = 1,15; CI 95%: 1,03–1,28; *p* = 0,02). Analyzing subgroups, we found that migraine was associated with both a moderate increased risk of all-cause dementia (OR = 1,26; *p* = 0,00; 95% CI: 1,13–1,40) as well as a moderate increased risk of Alzheimer’s disease (OR = 2,00; *p* = 0,00; 95% CI: 1,46–2,75). This association was significant in both case–control and retrospective cohort studies but not in prospective studies.

**Conclusions:**

Our study supports the presence of a link between primary headaches and dementia. However, in the subgroup analysis, only patients with migraine showed a moderate increase risk for all-cause dementia and for Alzheimer’s disease. Additional rigorous studies are needed to elucidate the possible role of primary headaches on the risk of developing cognitive impairment and dementia.

**Supplementary Information:**

The online version contains supplementary material available at 10.1007/s10072-023-07069-0.

## Introduction

The prevalence of dementia, which encompass a range of progressive and devastating neurodegenerative disorders, continues to increase worldwide, constituting a growing global public health issue [1]. Dementia represents an enormous clinical and economic burden for modern society, being a leading global cause of disability, institutionalization, and mortality [2]. As longevity is constantly increasing, economic and social costs of dementia threaten to overwhelm existing resources [3].

Dementia is a term used to describe an heterogeneous group of neurological disorders characterized by cognitive dysfunction affecting memory, critical thinking and social abilities severe enough to interfere with daily life [4]. Alzheimer’s disease (AD) is the most common cause of dementia. However, different conditions other than AD, like Frontotemporal dementia (FTD), Dementia with Lewy bodies (DLB) and Vascular Dementia (VaD), can cause cognitive impairment. The latter share many cognitive and pathological features with AD [5]. Frequently, patients with a diagnosis of AD present with different mixtures of brain pathologies, complicating both diagnosis, as well as treatment [6]. Therefore, the term Alzheimer’s disease and Related Dementias (ADRD) that encompasses neurodegenerative diseases causing dementia, is often used in epidemiological and pathological studies [7].

ADRD are multifactorial diseases caused by complex interplay of genetic and environmental factors. Recent studies estimate that more than a third of cases are potentially due to modifiable risk factors (e.g. hypertension, physical inactivity, smoking, hypercholesterolemia, overweight and obesity) [8, 9]. Identification of modifiable risk factors for dementia raises opportunity for both primary and secondary prevention plans, explaining the present growing interest in this field [10, 11].

Primary headaches, including migraine, tension-type headache and cluster headache, are one of the most prevalent neurological disorders worldwide [12, 13]. Recently, several epidemiological studies reported a positive association between primary headaches and AD as well as related dementias [14–17]. This association has been explained on the basis of the comorbidity between primary headaches and cardiovascular diseases, like hypertension and stroke, that are modifiable risk factors for dementia [18, 19]. However, conflicting results emerged in some studies. These findings may be due to differences in diagnostic criteria for cognitive deficits, in methodological strategies, and in the size of investigated populations [20].

The purpose of this study was to perform a systematic review and meta-analysis of the available data regarding association between primary headaches and dementia. In order to reduce previously observed heterogeneity, we investigated separately different primary headaches, and performed further subgroup analysis based on study design (case–control, retrospective and prospective cohort studies).

## Methods

### Data sources and searches

This study was conducted in accordance with the guidelines of the Preferred Reporting Items for Systematic Reviews and Meta-Analyses (PRISMA) consortium [21]. The protocol was registered in the International Prospective Register of Systematic Reviews (PROSPERO) platform (Registration ID: CRD42022380469).

*Pubmed*, *Scopus, Web of Science, Science Direct* and *BMC* were searched for articles investigating headache as possible risk factor for dementia of all causes. Search terms applied for literature search are reported in the Supplementary Material. No language, study design restrictions, or date of publication limit were applied. The search was conducted by one author (A.C.) up to September the 1st, 2022.

### Study selection

Only studies in English language, released on peer-reviewed journals involving human subjects with focus on primary headaches as a risk factor for dementia were considered for this systematic review. Reviews, case reports and interventional studies were excluded. Only studies conducted on patients with a diagnosis of primary headache and/or dementia, that investigated possible interactions or relations between the two conditions, were considered. Two blinded and independent investigators (A.C. and L.G.) screened for title and abstracts all the identified studies and excluded papers that did not meet the above-mentioned criteria. Subsequently, the same investigators reviewed the selected articles by full text and excluded papers that did not meet inclusion criteria. The final list of the included articles was then approved by an expert senior reviewer (F.R.).

### Data extraction

Investigators (A.G., F.F.) independently extracted data from the articles. The extracted data included: authors, study title, country where the study was conducted, year of publication, study design, sample size, duration of follow-up, age and gender distribution of participants, criteria used for headache and dementia diagnosis and adjusted confounders.

### Risk of bias assessment

The Newcastle–Ottawa scale (NOS) was used independently by two investigators (A.G., F.F.) to assess the quality of cohort studies [22]. Points were assigned (stars) for each article considering the following subsections: ranged from 0 to 9 points for cohort studies, participant selection and exposure measurement (0–4 stars), comparability (0–2 stars), outcome assessment and adequacy of follow up (0–3 stars). Higher the number of stars, higher the quality of the study. Scores of 0–3, 4–6, and 7–9 were considered to indicated low, moderate, and high quality, respectively.

### Statistical analysis

Statistical analysis was conducted using IBM SPSS Statistics 28.0 software. Distribution frequency of patients with primary headache with or without dementia was extracted for all the included studies. OR with 95% CI was then calculated. Random effect model was used to obtain a pooled measure of ORs (95% CI). Higgins’s I^2^ statistic was used to evaluate heterogeneity. We considered levels of I^2^ as follows: 25% low, 25 to 50% moderate, and above 75% high heterogeneity. Publication bias was evaluated through the analysis of asymmetry of the funnel plot and by using the Egger test. Finally, we underwent subgroup analysis stratifying the selected studies based on study design (case control, retrospective or prospective cohort studies), type of headache (primary headaches, migraine) and type of dementia (Alzheimer’s Disease, Vascular Disease). We could not perform other subgroup (e.g. other types of headache, other types of dementia) analysis due to lack of data.

## Results

### Literature search

**Fig. 1:**
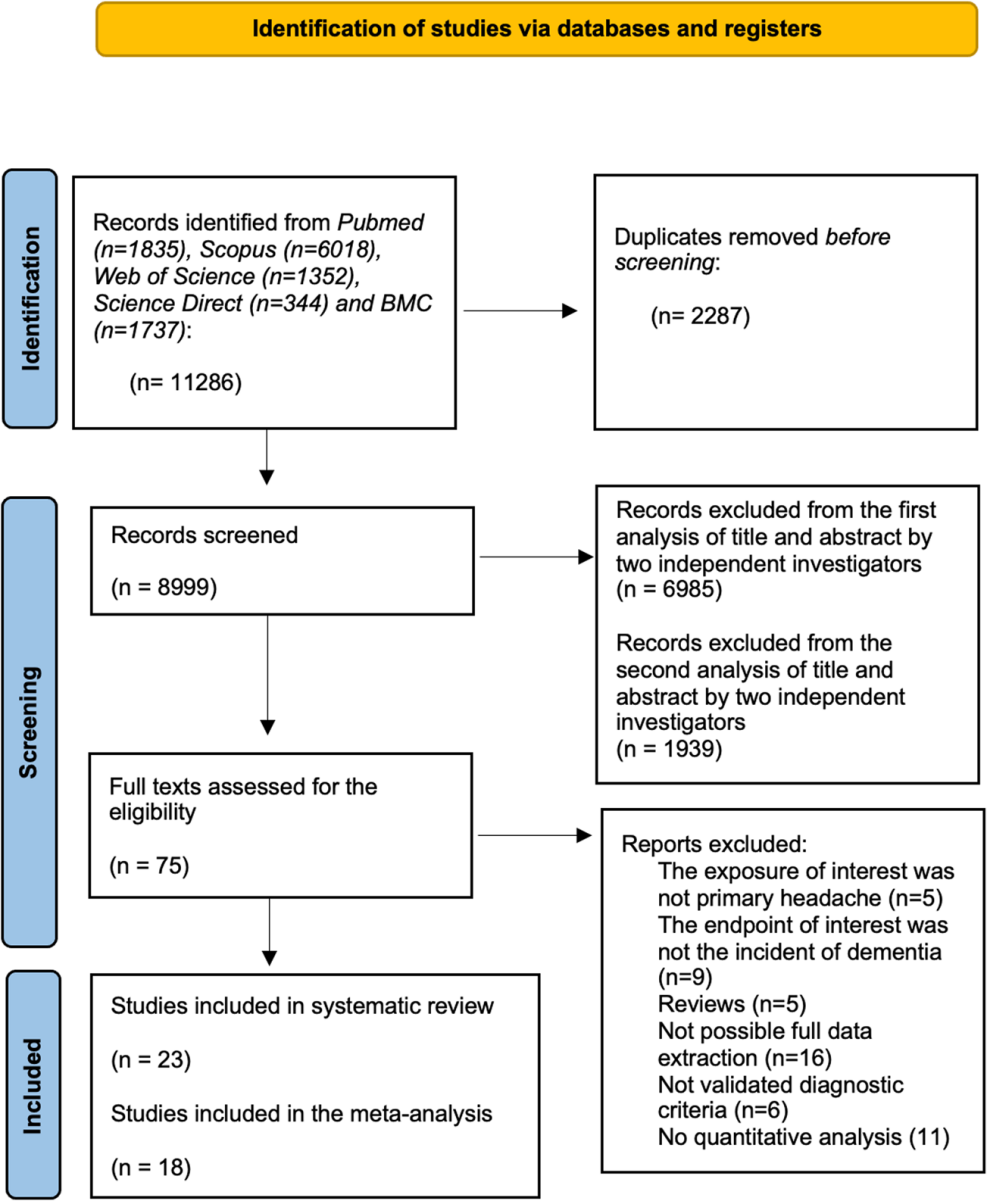
Flowchart PRISMA. Figure 1 shows the steps of our bibliographic search, edited following PRISMA guidelines

The initial search carried out in databases such as *Pubmed, Scopus, Web of Science, Science Direct* and *BMC* provided a total of 5926 bibliographical references subdivided as follows: *Pubmed* 1835, *Scopus* 6018, *Science Direct* 344, *Web of Science* 1352 and *BMC* 1737. After removing duplicates, 8999 papers were identified. A preliminary screening was carried out through the analysis of title and abstract. After a second screening, a total of 75 articles were selected. By applying inclusion criteria, 23 studies were then identified. Of these, 5 studies were excluded from statistical analysis for the following reasons: two studies did not show data from comparison group; three studies shared the same cohort population of another included article; one study displayed only measures of incidence. Finally, a total of 18 studies were selected for statistical analysis. Selection process pipeline is displayed in Fig. 1.

### Characteristics of the included studies and risk of bias analysis

The characteristics of the included studies are summarized in Table 1.

**Table 1:** Characteristics of the included studies

Author	Country	Study design	Follow-up (years)	% Female	Age	Headache type	Dementia type	Confounders adjusted
*Chuang -2013* [23]	China	Retrospective Cohort	12	71,3	42,2	Migraine	All cause of dementia	Age, sex diabetes, hypertension, depression, head injury and CAD
*Dewey – 1988* [24]	UK	Case–control	NA	NA	> 65	Headache	AD, all cause of dementia	Age and sex
*Echiverri – 2017* [25]	USA	Case–control	NA	58	75	Migraine, tension-type headache, unclassifiables	MCI, All cause of dementia, AD, VaD, LBD, FTD, unknown cause	Age, gender, and Mini-Mental Status Examination score
*George – 2020* [26]	USA	Prospective cohort	21	55,9	51–70	Migraine, non migraine headache	All cause of dementia	Age, sex, race-center, APOE4, income, education, BMI, smoking, hypertension, diabetes, CHD, drinking, HDL, cholesterol, and total cholesterol
*Hagen-2014* [27]	Norway	Prospective cohort	15	49,7	≥ 20	Migraine, Nonmigraine headache	AD, VaD and mixed dementia	Age, gender, education, smoking, SBP e DBP, antihypertensive medication, physical activity, BMI, cardiovascular disease, cholesterol, HADS score, abstention from alcohol, triglycerides, marriage, Glucose, reported stroke, angina
*Hurh – 2022* [28]	Korea	Retrospective cohort	17	66,1	55,3	Migraine	AD, VaD, other specified dementias, unspecified dementia	Sex, Age, Household income, residential area, registered disability, history of stroke, history of ischemic heart disease, history of diabetes mellitus, history of hypertension, history of antidepressant use, smoking status, BMI, Drinking
*Islamoska – 2020* [29]	Denmark	Case–control	6,9	70	31–58	Migraine	Unspecified dementia, AD, VaD, FTD, LBD	Birthdate, sex, country of origin, marital status, highest educational level, myocardial infarction, heart failure, peripheral vascular disease, cerebrovascular disease, pulmonary disease, connective tissue disorder, peptic ulcer, liver disease, diabetes, diabetes complications, paraplegia, renal disease, cancer, metastatic cancer, severe liver disease, and human immunodeficiency virus
*Kim – 2022* [30]	Korea	Prospective Cohort	8	46,8	55,5	Tension-type headache, Migraine, other type of primary headaches	AD, VaD, unspecified dementia	Age, sex, BMI, household income, smoking status, alcohol consumption, physical exercise, blood pressure, fasting serum glucose, total cholesterol, depression, sleep disorders, Parkinson’s disease, head injury, Charlson comorbity index
*Kostev- 2019* [31]	UK	Retrospective Cohort	19	72,9	67,7	Migraine	VaD, AD, unspecified dementia	Age, sex, index year, diabetes mellitus, hyperlipidemia, coronary heart disease, stroke including transient ischemic attack, depression, intracranial injury, mental and behavioral disorders due to the use of alcohol, epilepsy, Parkinson’s disease, and osteoporosis
*Lee- 2019* [32]	Korea	Case–control	5	68	> 60 years	Migraine	All cause of dementia, AD	Age, sex, income, region of residence, and past medical history of hypertension, diabetes, and dyslipidemia
*Lee – 2021* [33]	Korea	Retrospective cohort	NA	76	≥ 55 years	Migraine	All cause of dementia (no VaD), AD	Age, sex, residence, Househod income, hypertension, diabetes mellitus, stroke, chronic kidney disease, disorders of lipoprotein metabolism and other lipidemias
*Liang – 2022* [34]	Sweden	Prospective cohort	3–6	64	72	Migraine, other type of primary headaches	All cause of dementia	Age, sex, education, smoking, physical activity, alcohol consumption, hyper- tension, diabetes, cardiovascular diseases, use of medication, *APOE* genotype
*Lin – 2018* [35]	Taiwan	Retrospective Cohort	1	55	76,75	Headache	VaD, AD, other subtypes	Age, sex, education, marital status, geographic region and comorbidities
*Martins – 2020* [36]	Portugal	Prospective cohort	5	75	›50	Migraine, non migraine headache	MCI, all cause of dementia	Age, gender, hypertension, diabetes, smoking history and dyslipidemia), co-morbidities, and medication; sociodemographic data, subjective cognitive complaints and depressive symptoms, cognitive performance
*Morgan – 1994* [37]	UK	Case–control	4	NA	> 65	headaches	All cause of dementia	Age, sex, CAPE score
*Morton – 2019* [38]	Canada	Prospective Cohort	5	66	> 65	Migraine	All cause of dementia—AD—VaD	Education, age, gender, lifetime histories of migraine, depression, myocardial infarction, other heart conditions, stroke, diabetes and hypertension
*Pavlovic- 2013* [39]	USA	Prospective Cohort	1	NA	> 70 years	Migraine	All causes of dementia	Sex, education, ethnicity, APOE-e4 carrier status, baseline pain interference and pain severity
*Recchia- 2016* [40]	Italy	Prospective Cohort	3,9	72,7	> 80	Any Headache	All cause of dementia	Age, sex and education
*Røttereng – 2015* [41]	Norway	Retrospective cohort	2	60	55- 89	Migraine	All cause of dementia	Educational level, anxiety, depression (assessed by the Hospital Anxiety and Depression Scale), smoking (current, previous, or never), BMI, Systolic blood pressure, physical activity
*Tyas – 2001* [42]	Canada	Case–control	5	62,4	65–93	Headaches, migraine	All cause of dementia	Age, sex
*Tzeng – 2016* [43]	Taiwan	Retrospective cohort	10	68,04	20–54	Primary headaches	All causes of dementia	Gender, age group, geographical area of residence, urbanization level of residence and monthly income
*Yang- 2016* [44]	Taiwan	Retrospective cohort	8.14	66,7	≥ 20	Tension Type Headache	All cause of dementia	Sex, age, diabetes, dyslipidemia, hypertension, IHD, AF, HF, Stroke, depression, head injury, Parkinson's disease, migraine, COPD
*Yin – 2018* [45]	Taiwan	Retrospective Cohort	5	71,25	> 65	Primary headaches	All cause of dementia, AD, VaD	Age, sex, hypertension, diabetes mellitus, hyperlipidemia, IHDs, AF, TUD, alcoholism, obesity, PD, CVA, major depression, CKD, and CAI

No restriction for study design was applied. Of the 23 [23–45] included papers, 9 were retrospective cohorts, 8 were prospective cohorts and 6 were case–control studies. Only studies which considered all types of primary headaches and dementia were included. As expected for epidemiological reasons, a high percentage of female subjects was found in the patient population. The studies were conducted in the following countries: 4 Korea, 4 Taiwan, 3 UK, 3 USA, 2 Canada, 2 Norway, 1 China, 1 Denmark, 1 Italy, 1 Portugal, 1 Sweden.

In all studies correlation between the two diseases was adjusted on confounders such as age, sex, education, country of origin and/or residence and in many cases also based on the patient’s medical history (comorbidities such as hypertension, diabetes, heart disease or depression).

In the present meta-analysis, thought the analysis of risk of bias, quality was found to be similar when comparing case–control and cohort studies, although case–control studies showed greater in-between score discrepancy. All studies obtained maximum score in the "comparability" section. However, differences in scoring based on study design emerged in the "selection" category: case–control had a score of almost 4 stars while cohort studies had an average of 3 stars. The average score in the “exposure” category was 2.12 points. In this category, half of the articles did not obtain the star for the assessment of exposure; only one did not describe the response rate [25], two articles did not use the same method for cases and controls [24, 42]. Only four studies obtained a star in adequacy of the follow-up category due to the scarcity of information provided by the other included papers [27, 36, 38]. Nonetheless, follow up duration was considered adequate in ten studies (with a single exception) [35]. All studies obtained a star in the “outcome ascertainment” section. One paper obtained a total of five stars [25], so it is considered with a moderate quality, and it is the study in our sample with the lowest score. Two are the studies with a total of 9 stars and therefore with the best rating on the risk of bias [26, 43]. Table 1 of supplementary materials shows the methodological qualities of the studies with NOS criteria.

### Association between primary headaches and dementia

**Fig. 2:**
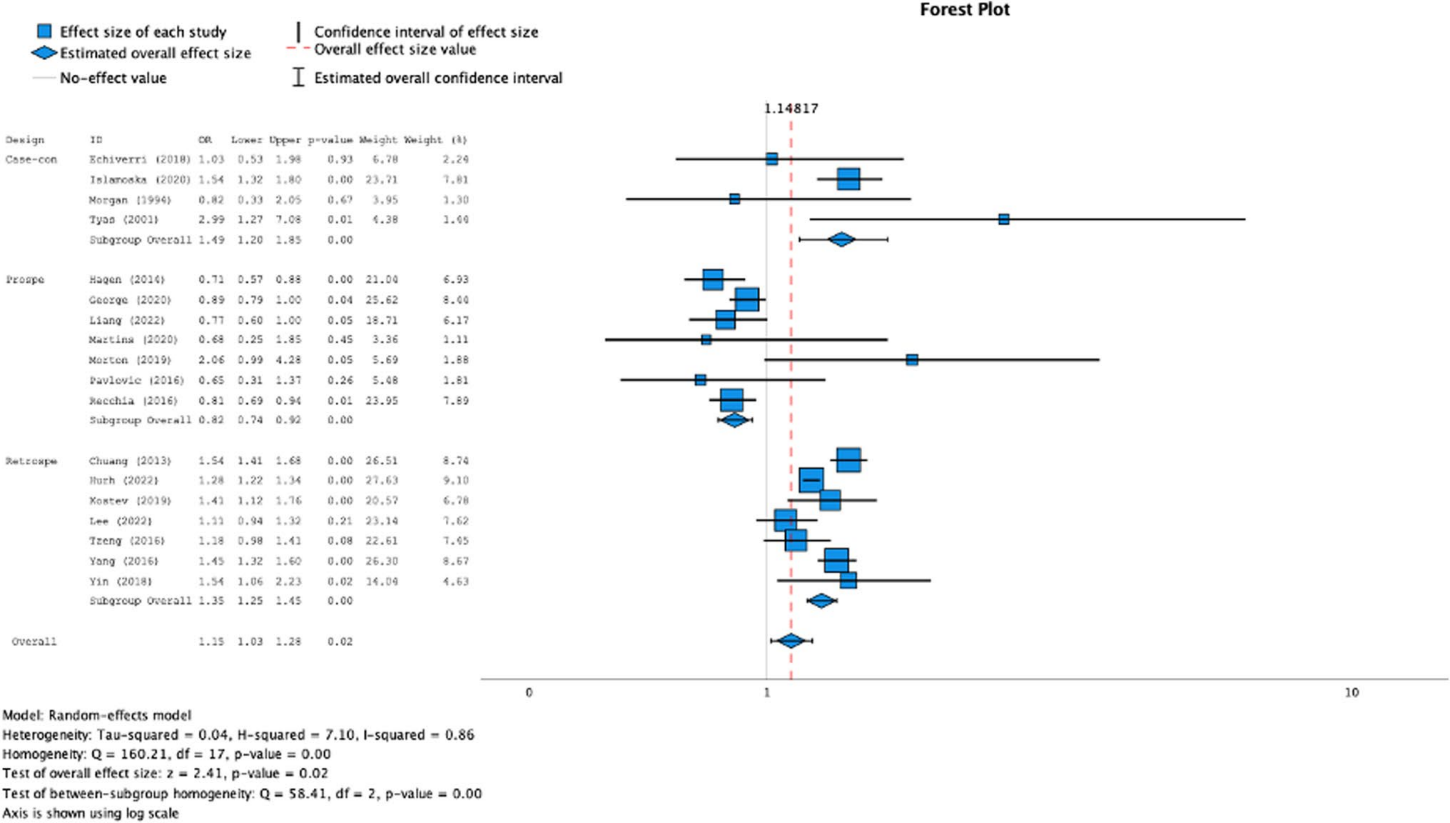
Forest plot of the association between headache ad dementia. Figure 2 shows the forest plot of the association between primary headache ad dementia, divided into 3 groups, based on study designs: case-controls, prospective cohorts, retrospective cohorts

**Fig. 3:**
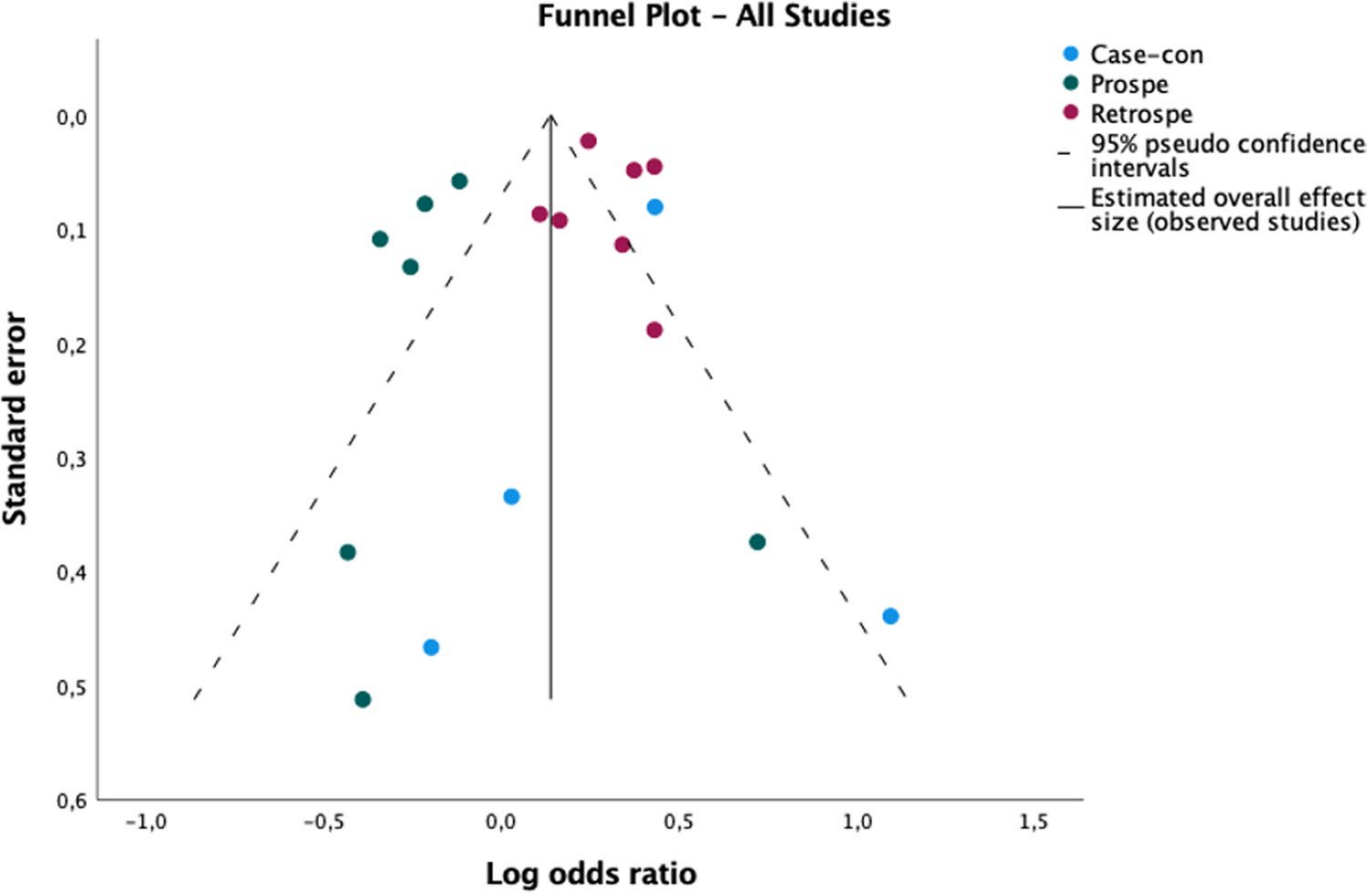
Funnel plot in the studies investigating the association between headache and dementia. Figure 3 shows the funnel plot of the studies investigating the association between primary headache and dementia, divided into 3 groups, based on study designs: case-controls, prospective cohorts, retrospective cohorts

We conducted a meta-analysis to investigate the risk of developing dementia (all cause) in people with a history of any type of primary headache. Eighteen articles were included [23, 25–31, 33, 34, 36–40, 42–45] in the statistical synthesis. A total of 203.042 patients with primary headaches and a total of 763.249 controls were included in the meta-analysis. The results of the pooling analysis (forest plot Fig. 2) showed that a previous diagnosis of any primary headache was associated with an increased risk of dementia (OR = 1,15; CI 95%: 1,03–1,28; *p* = 0,02). However, the analysis of I^2^ index showed high levels of heterogeneity (I^2^ = 85,9). The analysis of the asymmetry of the funnel plot (Fig. 3) and Egger’s Regression-based Test showed low risk of publication bias (Coefficient = 0,156; SE = 0,0822; t = 1,903; *p* = 0,075; 95% CI: -0,018–0,331).

To further clarify the high heterogeneity, we conducted a subgroup analysis according to the study design (case control, retrospective cohort, prospective cohort). Statistical analysis showed that both case studies (OR = 1,49; *p* = 0,00; 95% CI: 1,20–1,85; I^2^ = 9,2%) and retrospective cohort studies (OR = 1,35; *p* = 0,00; 95% CI: 1,25–1,45; I^2^ = 61,9%) were significant and the pooled effect sizes showed a moderate effect of the association between history of headache and risk of dementia, while prospective cohort studies were significant (OR = 0,82; *p* = 0,00; 95% CI: 0,74–0,92; I^2^ = 26,9%), but the relation between the two investigated variables was negative. After subgroup analysis, the heterogeneity in each group was considerably low (forest plot Fig. 2). In the other hand, publication bias (Fig. 3) remained low in both case–control and prospective cohort studies (*p* = 0,101 and *p* = 0,060), while appeared higher in retrospective cohort studies (*p* = 0,007).

### Association between migraine and dementia

**Fig. 4:**
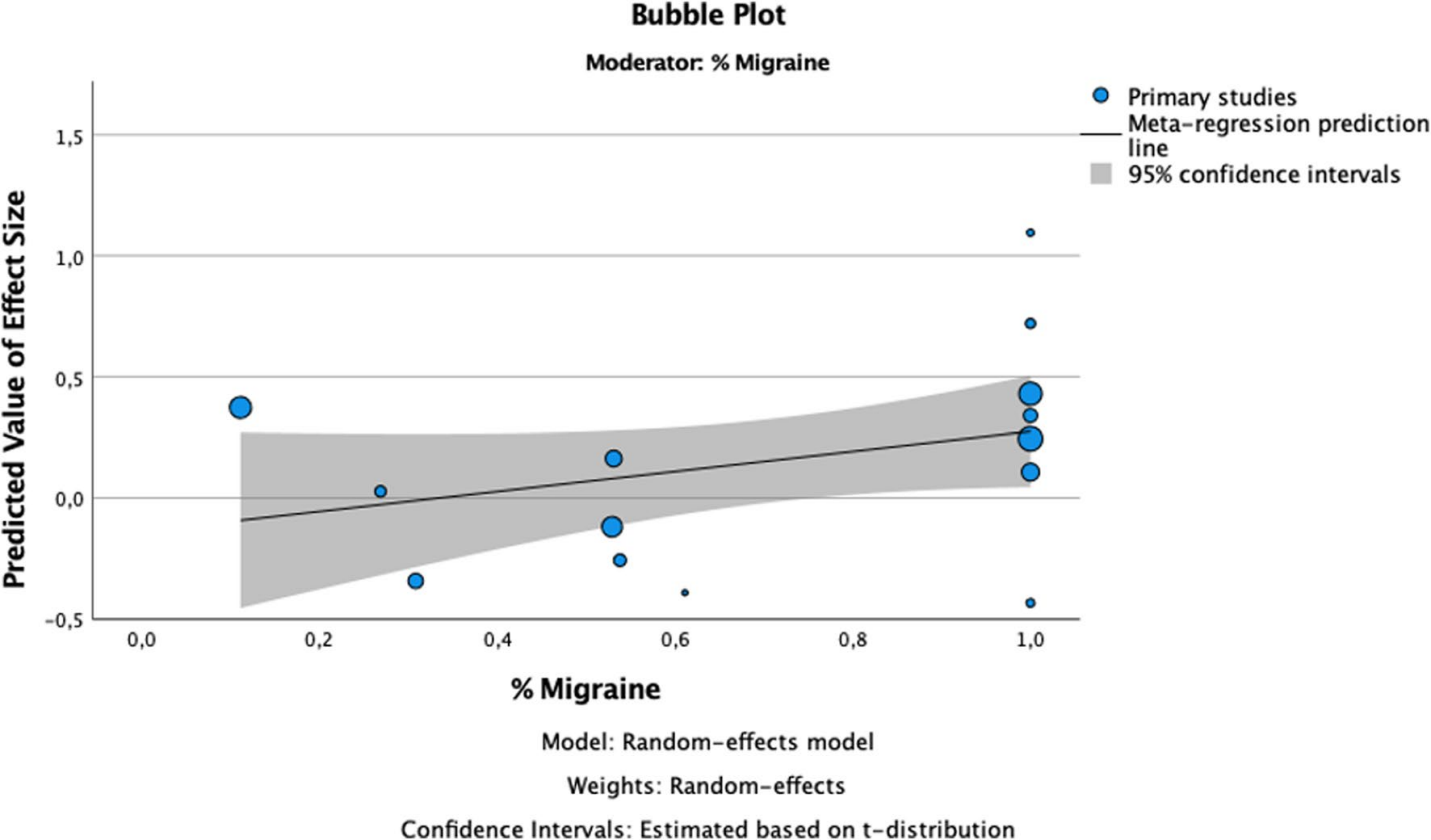
Moderator analysis. Figure 4 shows the conducted moderator analysis. We can observe significant direct relation between percentage of migraineurs patients and the effect size

**Fig. 5:**
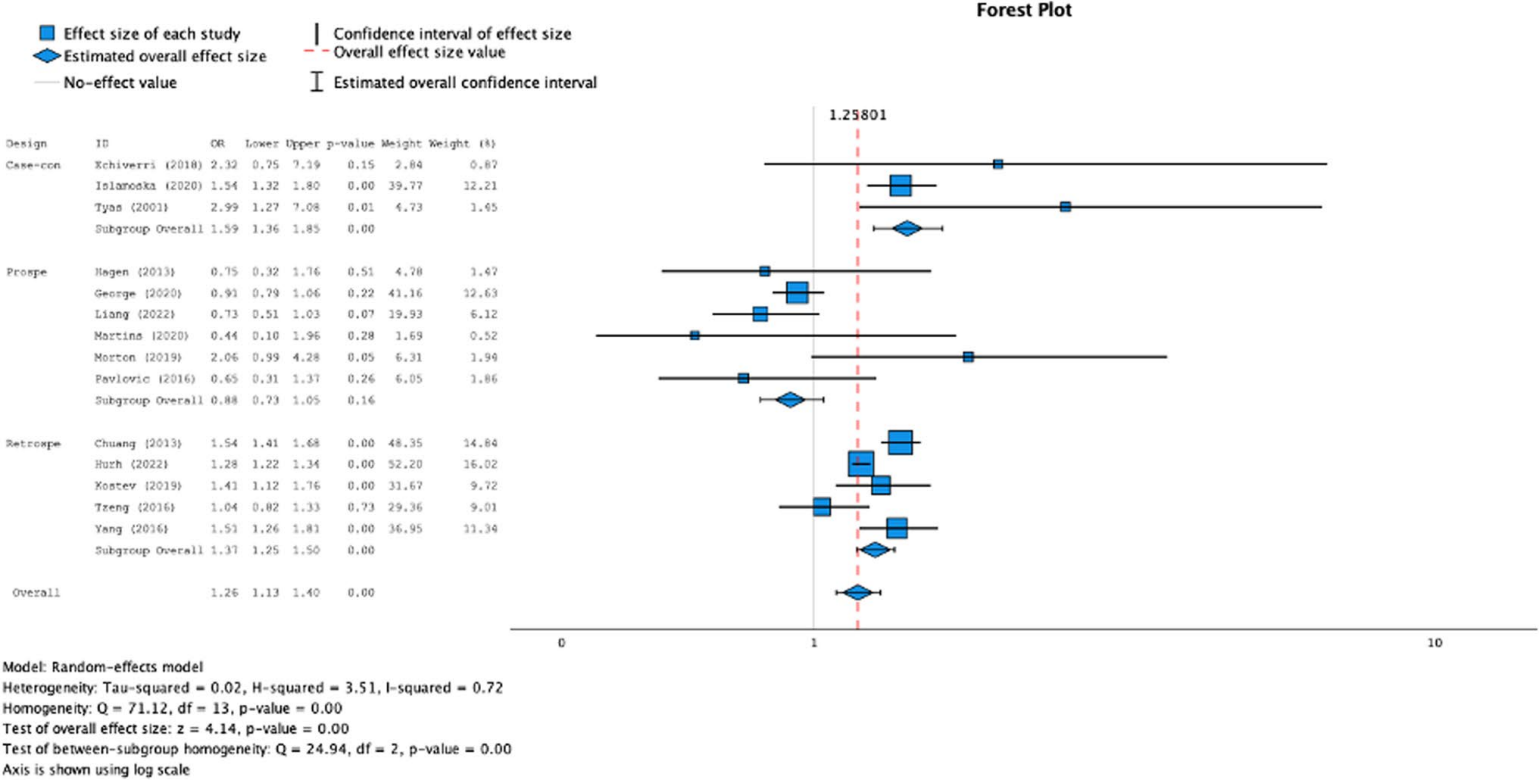
Forest plot of the association between migraine and dementia. Figure 5 shows the forest plot of the association between migraine and dementia, divided into 3 groups, based on study designs: case-controls, prospective cohorts, retrospective cohorts

**Fig. 6:**
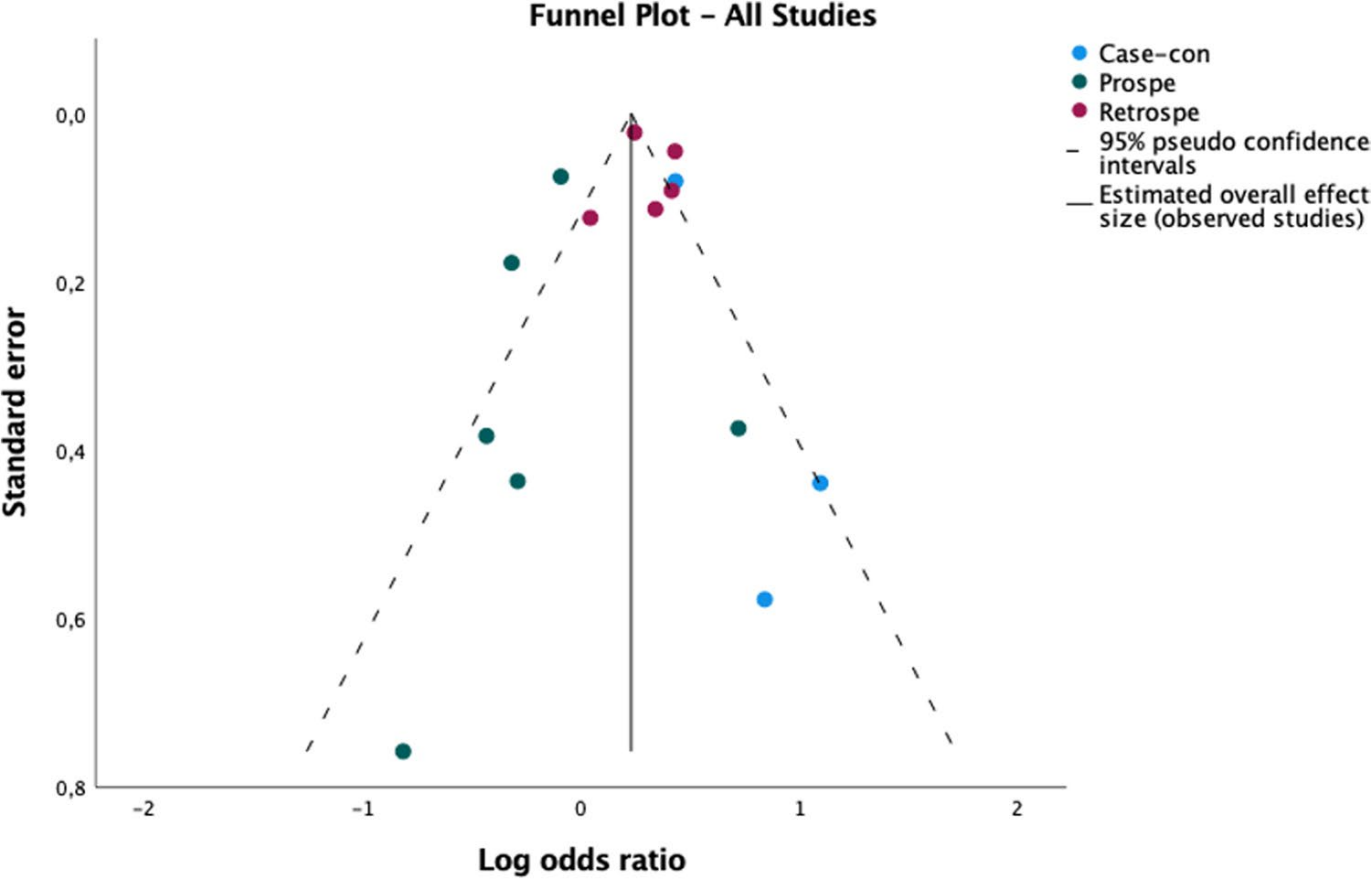
Funnel plot of the studies investigating the association between migraine and dementia. Figure 6 shows the funnel plot of the studies investigating the association between migraine and dementia, based on study designs: case-controls, prospective cohorts, retrospective cohorts

To evaluate also the risk of dementia in migraine we performed a sub-analysis of the studies reporting data about risk of dementia in migraineurs. We included sixteen studies [23, 25–31, 33, 34, 36, 38, 39, 42–44] in the meta-analysis. A total of 107.112 patients with migraine and a total of 346.376 controls were included in the meta-analysis. We calculated pooled effect size (forest plot Fig. 4) and results showed that migraine was associated with a moderate increased of risk of dementia (OR = 1,26; *p* = 0,00; 95% CI: 1,13–1,40) (Fig. 5). The analysis of I^2^ index showed high levels of heterogeneity (I^2^ = 71,5%) and high levels of publication bias (funnel plot Fig. 6; results of Egger’s Regression-based Test: Coefficient = 0,270; SE = 0,0772; t = 3,495; *p* = 0,004; 95% CI: 0,102–0,438).

Subgroup analysis (forest plot Fig. 4) based on study design showed significant pooled effect size in both case–control (OR = 1,59; *p* = 0,00; 95% CI: 1,36–1,85) and retrospective cohort studies (OR = 1,37; *p* = 0,00; 95% CI: 1,13–1,40) and revealed a moderate positive association between migraine and risk of dementia. The analysis of prospective cohort studies showed a negative association between the two variables, but this result was not statistically significant (OR = 0,88; *p* = 0,16; 95% CI: 0,73–1,05). Heterogeneity was moderate in retrospective cohort groups (I^2^ = 61,7%) and was low in both case–control and in prospective cohort group (I^2^ = 0%, I^2^ = 13,8% respectively). Finally, we found low levels of publication bias (funnel plot Fig. 5) in case-controls and prospective cohort studies (*p* = 0,219, *p* = 0,522 respectively) and high levels in retrospective studies (*p* = 0,020).

### Association between Alzheimer’s disease and headache

To evaluate the risk of Alzheimer’s Disease in patients with primary headache we performed a meta-analysis considering only studies reporting data about patients with Alzheimer’s Disease. We included seven studies [27, 28, 31, 33, 38, 44, 45] in the statistical analysis. We calculated pooled effect size (forest plot figure 1 supplementary materials) and results showed that headache was associated with increased of risk of Alzheimer’s disease (OR = 2,07; *p* = 0,00; 95% CI: 1,57–2,72). The analysis of I^2^ index showed high levels of heterogeneity (I^2^ = 85,4%) and high levels of publication bias (results of Egger’s Regression-based Test: Coefficient = 0,563; SE = 0,1900; t = 2,961; *p* = 0,031; 95% CI: 0,074–1,051) (funnel plot figure 2 supplementary materials).

To also evaluate the risk of Alzheimer’s Disease in migraine patients we performed a sub-analysis of the studies reporting data about risk of Alzheimer’s Disease in migraineurs. We included four studies [28, 31, 33, 38] in the meta-analysis. We calculated pooled effect size (forest plot figure 3 supplementary materials) and results showed that migraine was associated with a moderate increased of risk of Alzheimer’s Disease (OR = 2,00; *p* = 0,00; 95% CI: 1,46–2,75). The analysis of I^2^ index showed high levels of heterogeneity (I^2^ = 87,4%) and low levels of publication bias (results of Egger’s Regression-based Test: Coefficient = 0,284; SE = 0,2029; t = 1,400; *p* = 0,296; 95% CI: -0,589–1,157) (funnel plot figure 4 supplementary materials).

### Association between Vascular Dementia and headache

To evaluate the risk of Vascular Dementia in patients with primary headaches we performed a meta-analysis considering only studies reporting data about patients with Vascular Dementia. We included eight studies [27, 28, 31, 33, 38, 43–45] in the statistical analysis. We calculated pooled effect size (forest plot figure 5 supplementary materials) and results showed that headache was associated with increased of risk of Vascular Dementia (OR = 1,31; *p* = 0,00; 95% CI: 1,18–1,45). The analysis of I^2^ index showed low levels of heterogeneity (I^2^ = 0%) and high levels of publication bias (results of Egger’s Regression-based Test: Coefficient = 0,306; SE = 0,0832; t = 3,678; *p* = 0,010; 95% CI: 0,102–0,510) (funnel plot figure 6 supplementary materials).

To also evaluate the risk of Vascular Dementia in migraine patients we performed a sub-analysis of the studies reporting data about risk of Vascular Dementia in migraineurs. We included six studies [27, 28, 31, 33, 38, 43] in the meta-analysis. We calculated pooled effect size (forest plot figure 7 supplementary materials) and results showed that migraine was associated with a moderate increased of risk of Vascular Dementia (OR = 1,31; *p* = 0,00; 95% CI: 1,17–1,47). The analysis of I^2^ index showed low levels of heterogeneity (I^2^ = 0%) and high levels of publication bias (results of Egger’s Regression-based Test: Coefficient = 0,341; SE = 0,0870; t = 3,916; *p* = 0,017; 95% CI: 0,099–0,582) (funnel plot figure 8 supplementary materials).

## Discussion

In our meta-analysis we found that primary headaches are associated with a small increase in dementia risk. Analyzing subgroups of headache patients, we found that only migraine was associated with a moderate increased risk of both all-cause dementia and Alzheimer’s disease. This association was significant in both case–control and retrospective cohort studies but not in prospective studies. Our data are in accord with four previously published meta-analyses that found a similar increased risk for dementia in patients with a positive history for migraine [14–17]. However, in comparison with previous studies, we included a large sample of examined individuals and we examined both case–control and cohort studies showing than only case–control and retrospective cohort studies clearly support an association between migraine and dementia.

The association between migraine and dementia is still matter of intensive debate. In a recent paper, Vassallo et al. examined in details all the criticisms related to the studies that investigated such interesting but complex association [46]. First of all, frequently the term “headache” and “migraine” are used incorrectly and secondary headaches are often not ruled out. In our study, we selected patients with an ICHD-3 diagnosis of “primary headache” in order to avoid such misclassification. Then, the temporal distance between the onset of primary headaches and dementia may significantly influence the study of such association. Data collection regarding the frequency as well as the severity of migraine attacks is still a major problem for the study of the association. Finally, they observed that there is no clear biological explanation for the association between migraine and dementia.

Several, different pathogenetic mechanisms may explain the association observed between headache and dementia. First of all, many studies have clearly shown that patients with headaches are at significantly increased risk of developing depression [47] while depression is a well-known risk factor for cognitive impairment and dementia [48], and this association is explained by an overlap of common genetic risk factors [49]. It is of interest to note that subjects carrying mutations in the presenilin-1 gene, a gene responsible of early-onset Alzheimer’s disease, frequently complained of headache even in the pre-symptomatic phase of the disease [50].

Then, both primary headaches and dementia have been associated with several vascular risk factors, such as hypertension, diabetes and dyslipidemia [51, 52]. Altered insulin resistance have been described in both primary headaches [53] and dementia [54] and may represent a common link between these diseases. Of particular interest is the recently developed concept of brain insulin resistance, a condition characterized by altered insulin signaling in the central nervous system. Insulin receptors have been found ubiquitously in the brain and their expression is high in select regions such as the cerebellum, cortex, and hypothalamus. Insulin influences cerebral metabolism, increases turnover of neurotransmitters, such as dopamine, and its signaling is important for mitochondrial functioning. Therefore, altered insulin transport across the blood brain barrier as well as altered insulin receptor expression may explained the increased risk of developing cognitive impairment in patients suffering from primary headaches.

Finally, the presence of neuroinflammation is a common feature of dementia. Reactive microgliosis, release of several proinflammatory cytokines, oxidative damage and mitochondrial dysfunction are associated with the pathogenesis of Alzheimer’s disease and related dementia [55]. Recently, the role of neuroinflammation has been investigated also in patients with migraine. Several cytokines, including tumor necrosis factor alpha, interleukin 1, and adiponectin, have been implicated in the pathogenesis of migraine. In addition, studies in experimental animals have demonstrated that immunological responses are involved in the pathogenesis of migraine [56]. Therefore, repeated headache attacks may activated neuroinflammatory mechanisms predisposing the brain to neurodegeneration.

Our study has certain limitations. While the diagnoses of primary headaches or migraine relates to the well validated criteria of the International Headache Society [57], several different diseases may be characterized by dementia. For example, the diagnosis of Alzheimer’s disease may be performed using only clinical [58] or biological [59] criteria. Therefore, the clear association with a specific dementia subtype still needs additional studies. Furthermore, we did not include covariate analysis in this meta-analysis. However, the number of patients included in the meta-analysis is very high, making the conclusions of this study reliable and supporting the need of additional investigations.

In conclusion, our study further supports the presence of a significant association between primary headaches and the risk for developing dementia in advanced age. This association seems to be of particular relevance between migraine and Alzheimer’s disease. However, relationship between primary headaches and dementia needs further, detailed investigations. Indeed, the fact that most of the included prospective studies do not confirm this association might raise the suspicion of the existence of confounding variables.

### Supplementary Information

Below is the link to the electronic supplementary material.

Supplementary file1 (DOCX 353 KB)

## Data Availability

All collected data are available upon request to corresponding author.
