# Supplementary material for: Is headache a risk factor for dementia? A systematic review and meta-analysis
Source: Neurol Sci. 2023 Sep 18;45(3):1017–30. doi: 10.1007/s10072-023-07069-0 (PMC10858119; doi:10.1007/s10072-023-07069-0)
Supplement: Supplementary file 1 — Supplementary file1 (DOCX 353 KB) [file 10072_2023_7069_MOESM1_ESM.docx]

**Supplementary materials**

**Search Terms**

*Pubmed*, *Scopus, Web of Science, Science Direct* and *BMC* were searched applying the following search strategy:

*((headache)* OR *(primary headaches)* OR *(migraine))* AND *((dementia)* OR *(Alzheimer)* OR *(Alzheimer’s disease)* OR *(Frontotemporal dementia)* OR *(Lewy body dementia)* OR *(vascular dementia))*

**Table 1**

**“Analysis of Risk of Bias”**

The Newcastle-Ottawa Scale (NOS) for assessing the quality of nonrandomised studies in meta-analyses was used to analyze risk of bias in this study.

Scores of 0–3 stars indicate low quality, 4–6 stars moderate quality, and 7–9 stars hight quality.

| AUthor | SELECTION | COMPARABILITY | EXPOSURE | OUTCOME | TOT |
| --- | --- | --- | --- | --- | --- |
| Lin-2018 | 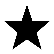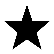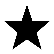 | 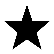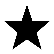 |  | 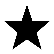 | 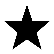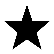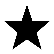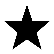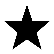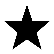 |
| Morton-2019 | 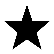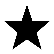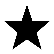 | 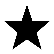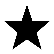 |  | 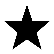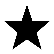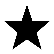 | 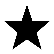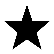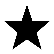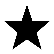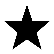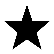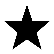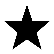 |
| Kostev-2019 | 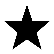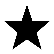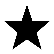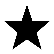 | 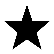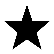 |  | 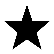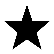 | 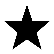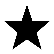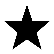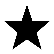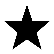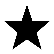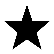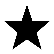 |
| Pavlovic-2013 | 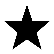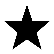 | 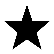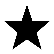 |  | 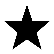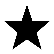 | 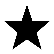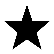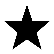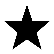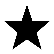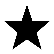 |
| George-2020 | 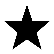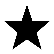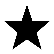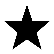 | 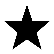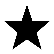 |  | 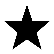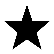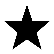 | 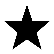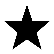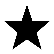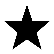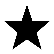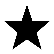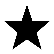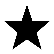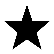 |
| Martins-2020 | 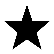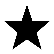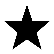 | 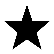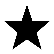 |  | 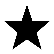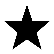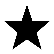 | 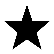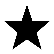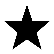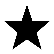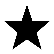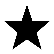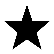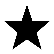 |
| Recchia-2016 | 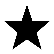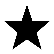 | 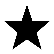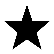 |  | 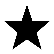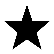 | 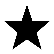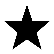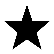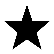 |
| Yang-2016 |  |  |  |  |  |
| Hagen-2013 |  |  |  |  |  |
| Chuang-2016 |  |  |  |  |  |
| Yin-2018 |  |  |  |  |  |
| Hurh – 2022 |  |  |  |  |  |
| LIANG – 2022 |  |  |  |  |  |
| LEE – 2021 |  |  |  |  |  |
| KIM - 2022 |  |  |  |  |  |
| Røttereng - 2015 |  |  |  |  |  |
| Echiverri-2017 |  |  |  |  |  |
| Morgan-1994 |  |  |  |  |  |
| Tyas-2001 |  |  |  |  |  |
| Dewey-1988 |  |  |  |  |  |
| Tzeng-2016 |  |  |  |  |  |
| Lee-2019 |  |  |  |  |  |
| Islamoska-2020 |  |  |  |  |  |

Figure 1

“Forest plot of the association between primary headaches and Alzheimer’s Disease”

Figure 2

“Funnel plot in the studies investigating the association between headache and Alzheimer’s Disease”

Figure 3

“Forest plot of the association between migraine and Alzheimer’s Disease”

Figure 4

“Funnel plot in the studies investigating the association between migraine and Alzheimer’s Disease”

Figure 5

“Forest plot of the association between Primary headaches and Vascular Dementia”

Figure 6

“Funnel plot in the studies investigating the association between headaches and Vascular Dementia”

Figure 7

“Forest plot of the association between migraine and Vascular Dementia”

Figure 8

“Funnel plot in the studies investigating the association between migraine and Vascular Dementia”
